# The Salt Sensitivity Induced by Disruption of Cell Wall-Associated Kinase 1 (*SlWAK1*) Tomato Gene Is Linked to Altered Osmotic and Metabolic Homeostasis

**DOI:** 10.3390/ijms21176308

**Published:** 2020-08-31

**Authors:** Victoriano Meco, Isabel Egea, Ana Ortíz-Atienza, Stéphanie Drevensek, Elisabeth Esch, Fernando J. Yuste-Lisbona, Fredy Barneche, Wim Vriezen, María C. Bolarin, Rafael Lozano, Francisco B. Flores

**Affiliations:** 1 Department of Stress Biology and Plant Pathology, Centro de Edafología y Biología Aplicada del Segura, CSIC, Campus Universitario de Espinardo, 30100 Murcia, Spain; iegea@cebas.csic.es (I.E.); mbolarin@cebas.csic.es (M.C.B.); borjaflores@cebas.csic.es (F.B.F.); 2 Centro de Investigación en Biotecnología Agroalimentaria (BITAL). Universidad de Almería, 04120 Almería, Spain; anaortiz@ual.es (A.O.-A.); fyuste@ual.es (F.J.Y.-L.); rlozano@ual.es (R.L.); 3 Institut de Biologie de l’École Normale Supérieure (IBENS), Paris Sciences et Lettres Research University, F-75005 Paris, France; stephanie.drevensek@universite-paris-saclay.fr (S.D.); barneche@biologie.ens.fr (F.B.); 4 BASF Vegetable Seeds, Napoleonsweg 152, 6083AB Nunhem, The Netherlands; elisabeth.esch@vegetableseeds.basf.com (E.E.); wim.vriezen@vegetableseeds.basf.com (W.V.)

**Keywords:** *Solanum lycopersicum*, insertional mutant, osmotic stress, ion stress, metabolites, fruit yield

## Abstract

Tomato cell wall-associated kinase 1 (*SlWAK1*) has only been studied in biotic stress response and hence its function in abiotic stress remains unknown. In a screening under salinity of an insertional mutant collection of tomato (*Solanum lycopersicum* L.), a mutant exhibiting lower degree of leaf chlorosis than wild type (WT) together with reduced leaf Na^+^ accumulation was selected. Genetic analysis of the mutation revealed that a single T-DNA insertion in the *SlWAK1* gene was responsible of the mutant phenotype. *Slwak1* null mutant reduced its shoot growth compared with WT, despite its improved Na^+^ homeostasis. *SlWAK1* disruption affected osmotic homeostasis, as leaf water content was lower in mutant than in WT under salt stress. In addition, *Slwak1* altered the source-sink balance under salinity, by increasing sucrose content in roots. Finally, a significant fruit yield reduction was found in *Slwak1* vs. WT under long-term salt stress, mainly due to lower fruit weight. Our results show that disruption of *SlWAK1* induces a higher sucrose transport from source leaf to sink root, negatively affecting fruit, the main sink at adult stage.

## 1. Introduction

Salt stress is responsible for reduced crop growth and the cause of important economic losses in agricultural production. Therefore, it is a priority to develop new crop varieties able to maintain yield under stress conditions. Tomato (*Solanum lycopersicum* L.) is one of the most important succulent fruit bearing species in agriculture, and additionally, it has become a model species in plant research [1]. Despite the economic relevance of tomato, the mechanisms that govern responses to abiotic stresses in this horticultural species are not well characterized, and only a very small number of genes playing key roles in tomato tolerance to salinity throughout its life cycle have so far been identified [2,3,4]. To understand the physiological mechanisms responsible for salinity tolerance, it is important to bear in mind that plants respond to salinity by using two main mechanisms: a mechanism of osmotic tolerance to avoid the osmotic effect of the salt concentration outside the roots, and a mechanism of ionic tolerance to avoid the toxic effect of salt in the plant. To understand plant growth reduction induced by salinity it is necessary to take into account the energy costs of salinity tolerance. Interestingly, in the salt-tolerant wild tomato species *Solanum pennellii* we observed that endogenous Na^+^ concentration increased rapidly after applying salt stress in order to maintain the osmotic homeostasis and avoid the yield penalties [5], while the high ion exclusion in tomato induced by higher use of organic solutes to re-establish osmotic homeostasis occurs at the cost of plant growth [6]. Thus, the use of inorganic solutes seems an energetically effective way that does not compromise plant growth, while ion exclusion requires a higher use of organic solutes and, therefore, high energy costs because of their biosynthesis [7]. In conclusion, tomato tolerance to salt stress is not the result of only one mechanism; the osmotic homeostasis may be the predominant mechanism to avoid a high energetic cost, but in other circumstances it may be ion homeostasis to avoid Na^+^ toxicity the predominant mechanism.

Cell wall-associated protein kinases (WAKs) are receptor-like kinases localized in the plasma membrane with a cytoplasmic serine/threonine kinase domain and an extracellular domain that tightly binds to the pectin fraction of the cell wall but also to pectin fragments. In Arabidopsis WAK proteins are required for cell expansion and stress responses via their binding to pectin, in order to activate an intracellular signal transduction pathway via phosphorylation that finally induces changes in regulation of solutes contents [8,9]. It is well known that changes in concentration of solutes are involved in turgor regulation, a key parameter that needs to be tightly regulated during cell expansion and stress response. In tomato, there are seven predicted *WAK* genes and four of them occur in a clustered gene family that includes *SlWAK1*, located on chromosome 9 [10]. The wide divergence among *WAK* sequences prevented assignment relationships between tomato *WAK* genes and *Arabidopsis thaliana* orthologs, therefore the gene was named *SlWAK1*. In tomato, *SlWAK1* has been shown to be involved in biotic stress tolerance and silencing of *SlWAK1* compromised the plant immune response to bacterial infection resulting in enhanced disease symptoms [10]. However, much remains to be learned about the molecular mechanisms as Zhang et al. [11] has recently pointed out. In fact, a clear demonstration of the role of *WAKs* in host defence by loss-of-function analyses has been hindered by lethality or developmental defects of some *WAK* mutants and possibly redundancy.

Tomato mutants not only constitute much valuable genetic sources for breeders but their analysis is one of the most powerful genomic tools for the identification of genes involved in salt tolerance. Insertional mutagenesis has the advantage that mutated genes are tagged by an inserted element (transposon or T-DNA), allowing a speedy and effective way to fulfil its genomic localization and cloning. We have generated a collection of tomato (*S. lycopersicum*) insertional mutants in cultivar Moneymaker [12], where some of them have already been identified as being affected in salt tolerance [2,3]. Within this collection, we identified an insertional mutant containing a single T-DNA insertion localized in the promoter region of *SlWAK1*, causing the knockout of the tagged gene. Here we demonstrate that *SlWAK1* is required to re-establish the osmotic homeostasis and for sucrose transport balance between source leaves and sink roots.

## 2. Results

### 2.1. Molecular and Genetic Characterization of the Slwak1 Mutant

**Figure 1:**
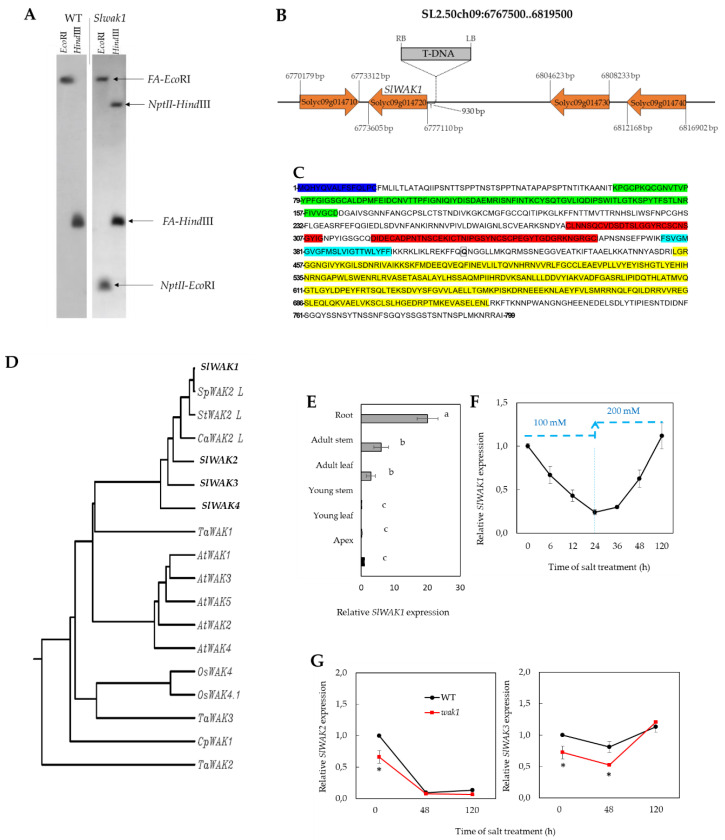
Identification of the knockout tomato mutant *Slwak1*. (**A**) Southern blot analysis to determine the number of T-DNA insertions in *Slwak1* mutant using a pool of T_2_ plants with a chimeric probe that includes the complete coding sequence of the *NptII* gene fused to 811 pb of the coding sequence of *FALSIFLORA* (*FA*) (used as hybridization positive control). Single restriction fragments observed in genomic DNA digested with *EcoRI* and *HindIII* indicate the presence of a single T-DNA insertion in the *Slwak1* genome. (**B**) Genomic organization of the WAK cluster in the tomato chromosome 9, composed of four isogenes coding for WAK proteins, and localization of the T-DNA insertion. *SlWAK1* gene is tagged by the T-DNA localized 930 bp upstream of the coding region, most probably in the promoter region of this gene. (**C**) Amino acid sequence predicted from the *SlWAK1* gene coding sequence, with predicted conserved protein domains, N-terminal signal peptide and transmembrane region highlighted, identified by making use of diverse bioinformatics tools (see Materials and Methods). In yellow the Ser/Thr kinase catalytic domain (STKc, 455–722 residues), in red the two calcium-binding EGF-like domains (EGF-Ca, 321–364 and 285–311 residues), in green the cell wall-associated receptor kinase galacturonan-binding Cys-rich domain (GUB-WAKb, 65–164 residues), in magenta the N-terminal signal peptide that targets the protein to the secretory pathway (1–15 residues) and in blue the transmembrane domain (377–399 residues). Boxed amino acid (glutamine, position 414) indicated the position where a premature stop codon is generated by a SNP in 12,007 EMS line. (**D**) Phylogenic tree constructed using rooted tree with branch length (UPGMA), making use of the GenomeNet bioinformatics tools (https://www.genome.jp/tools-bin/clustalw), based on neighbor-joining method after sequences alignment with ClustalW. The homologous sequences of *SlWAK1* were retrieved from NCBI and SGN databases or the scientific literature (see Materials and Methods). (**E**) Spatial expression pattern of *SlWAK1* gene in WT plants with six developed leaves grown in control conditions. (**F**) Time-course analysis of expression of *SlWAK1* in roots of WT plants salt-treated with 100 mM NaCl during 24 h and subsequently with 200 mM NaCl from 24 h to 120 h. (**G**) Expression of two flanking genes *SlWAK2* and *SlWAK3* in roots of WT and *Slwak1* plants after 0, 48 and 120 h of salt treatment (200 mM NaCl). In (**E**), (**F**) and (**G**), values are the mean ± SE of two independent assays, each with three biological replicates. Different lowercase letters in (**E**) indicate significant differences (LSD test, *p <* 0.05). Asterisks in (**G**) indicate significant differences between mean values of WT and *Slwak1* (Student’s *t*-test, *p <* 0.05).

During the screening for salinity tolerance of segregating populations (T_2_) coming from a T-DNA collection generated in tomato (*S. lycopersicum* L. cv Moneymaker), a mutant line was selected for its lower degree of leaf chlorosis compared with wild type (WT) plants, suggesting that this might be a salt tolerant mutant. Southern blot analysis revealed the presence of a single T-DNA copy in the mutant line (Figure 1A). The flanking sequences of the T-DNA insertion were cloned by anchor-PCR and these were compared with the tomato genome sequences available in the SOL genomics network database (https://solgenomics.net). The comparison of sequences allowed the localization of the T-DNA insertion on chromosome 9, positioned 930 bp upstream of the coding sequence Solyc09g014720. This sequence codes for a member of the family of plasmatic membrane receptors known as cell wall-associated kinase receptors (WAKs). The tagged gene was identified by Rosli et al. [10] and denominated *SlWAK1*, therefore the mutant line was denominated *Slwak1* after the annotation of the gene.

The genomic region (SL2.50ch09:6767500.6819500) where the T-DNA insertion was detected contains a cluster of four genes annotated as *WAK* genes (Figure 1B). These four genes share high sequence homology (Solyc09g014710, Solyc09g014720, Solyc09g014730 and Solyc09g014740) (identity score > 65%) and are likely derived from duplication events (paralogous genes). The tagged gene has a length of 3506 bp and consisted of four exons transcribed in a 2400-bp mRNA. The *SlWAK1* gene encodes a protein of 799 amino acids, with predicted pI of 6.13 and a MW of 88.3 KDa. The analysis of the protein sequence confirms the presence of all characteristic domains of WAK proteins: a Ser/Thr kinase catalytic domain (STKc, 455–722 residues), two repeated calcium-binding EGF-like domains (EGF-Ca, 321–364 and 285-311 residues), and a cell wall-associated receptor kinase galacturonan-binding Cys-rich domain (GUB-WAKb, 65–164 residues) (Figure 1C). An N-terminal signal peptide targeting the protein to the secretory pathway (1–15 residues, D-score of 0.861) and a transmembrane domain (377–399 residues, total probability 0.8548) are predicted in the protein sequence of SlWAK1 (Figure 1C). In sum, the tagged gene encodes all characteristic protein domains of WAK-coding genes. In fact, the multiple sequence alignment of the amino acid sequences encoded by the four genes of the cluster reveals that all of them have the above mentioned characteristic WAK domains conserved. The only difference regarding these conserved domains is that SlWAK4 has only one EGF-Ca domain instead of two like the other three *SlWAK* gene products of the cluster (Figure S1 (Supplementary Materials)).

Multiple sequence alignment and phylogenetic analysis of SlWAK1 protein alongside those of the other tomato WAK-coding genes within the cluster and others from the protein and genomic databases (*A. thaliana*, rice, wheat, the resurrection plant *Craterostigma plantagineum*), including several Solanaceae (potato, pepper) and a close tomato wild-relative species (*S. pennellii*), showed that WAK proteins from Solanaceae species are clustered in one group (Figure 1D). It is interesting to point out that maximum similarity of SlWAK1 is found with SlWAK2-like protein from the tomato wild relative species *S. pennellii*, and next with potato and pepper, being the degree of identity with these sequences higher (>84%) than with protein sequences from the other tomato WAK genes found in the genomic cluster of *SlWAK1* (*SlWAK2*, *SlWAK3* and *SlWAK4*) (65–80%). *A. thaliana* WAK proteins forms another cluster in the phylogenetic analysis and the relatively high degree of divergence observed between WAK-coding genes from *A. thaliana* and from tomato does not allow to establish a relationship of homology between *SlWAK1* and *A. thaliana WAK* genes (Figure 1D).

**Figure 2:**
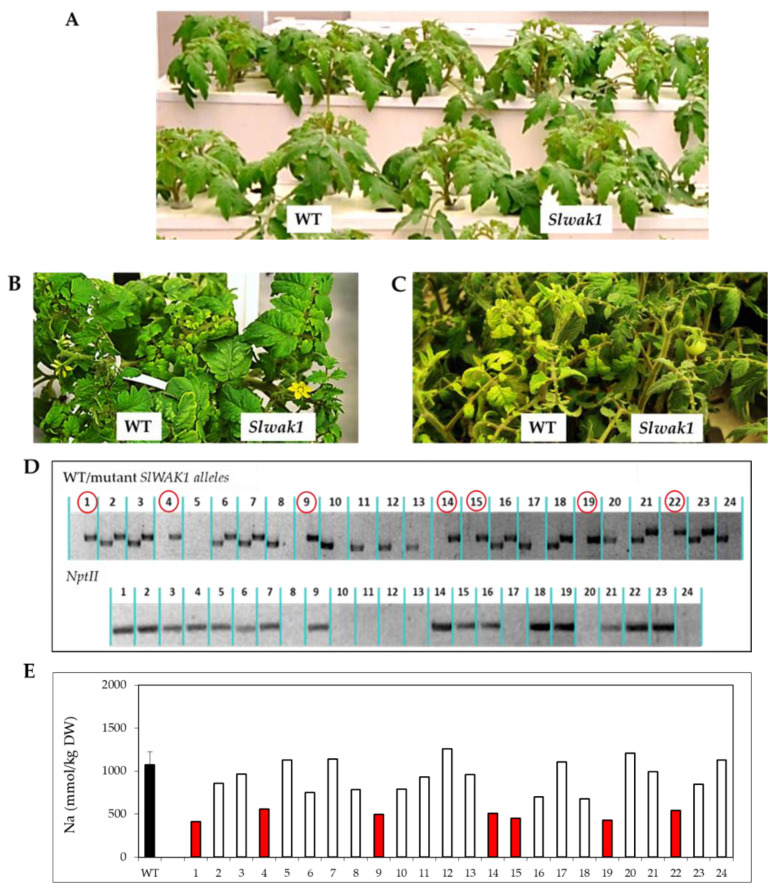
Phenotypic, physiological and genetic characterization of *Slwak1* mutant. At the phenotypic level, no differences between WT and the segregating progeny of *Slwak1* mutant plants are observed under control conditions (**A**), while the mutant plants showed lower degree of chlorosis in their leaves after 10 days (**B**) and 20 days (**C**) of 200 mM NaCl treatment. (**D**) PCR genotyping for presence of mutant (*Slwak1*) and WT *SlWAK1* alleles as well as *NptII* gene in a T_2_ *Slwak1* population. Individuals marked with red circles exhibited the mutant phenotype (with the mutant allele in homozygosis) and all of them showed presence of *NptII* gene. (**E**) Na^+^ accumulation in the first developed leaves of WT and T_2_ *Slwak1* population. The phenotype of the mutant plants (lower degree of leaf chlorosis under salinity) correlated with the genotype of the segregating progeny, as well as with lower Na^+^ accumulation in leaves of these plants under salt stress.

An interesting characteristic for selection of mutants affected in salt tolerance is that their phenotypic and growth characteristics were similar to WT before applying salt stress, as observed in the segregating population of *Slwak1* plants (Figure 2A). Under salt stress (200 mM NaCl), the T_2_ population showed different degree of leaf chlorosis after 10 days and, especially, after 20 days of salt treatment (Figure 2B,C). Homozygous plants for the mutation were identified by genotyping mutant and WT *SlWAK1* alleles and by amplification of *NptII* marker gene by PCR (Figure 2D). From 24 individuals of the segregating population, a correlation between the mutant phenotype and the genotype of T_2_ plants was observed since only the seven individuals with the mutant allele in homozygosis showed a lower leaf chlorosis compared with WT, and this coincided with presence of *NptII* marker gene.

Hence, the phenotype segregation observed (17 WT:7 mutant) was consistent with a monogenic recessive inheritance for the *Slwak1* mutant (Χ^2^ = 0.22, *p* > 0.5). Moreover, Na^+^ accumulation in the first two developed leaves also co-segregated with the genotype of T_2_ plants grown under salt stress (Figure 2E). *Slwak1* mutant was identified by its phenotype of lower degree of leaf chlorosis compared with WT when grown in salinity; this chlorosis is a consequence of the accumulation of Na^+^ due to salt stress and the T_2_ homozygous plants identified by genotyping exhibited such mutant phenotype and the lowest Na^+^ accumulation in leaves compared with WT and hemizygous and azygous plants. The identified homozygous T_2_ *Slwak1* plants were grown in a greenhouse to obtain the homozygous T_3_ lines that have been used for characterization of the *Slwak1* mutant.

**Figure 3:**
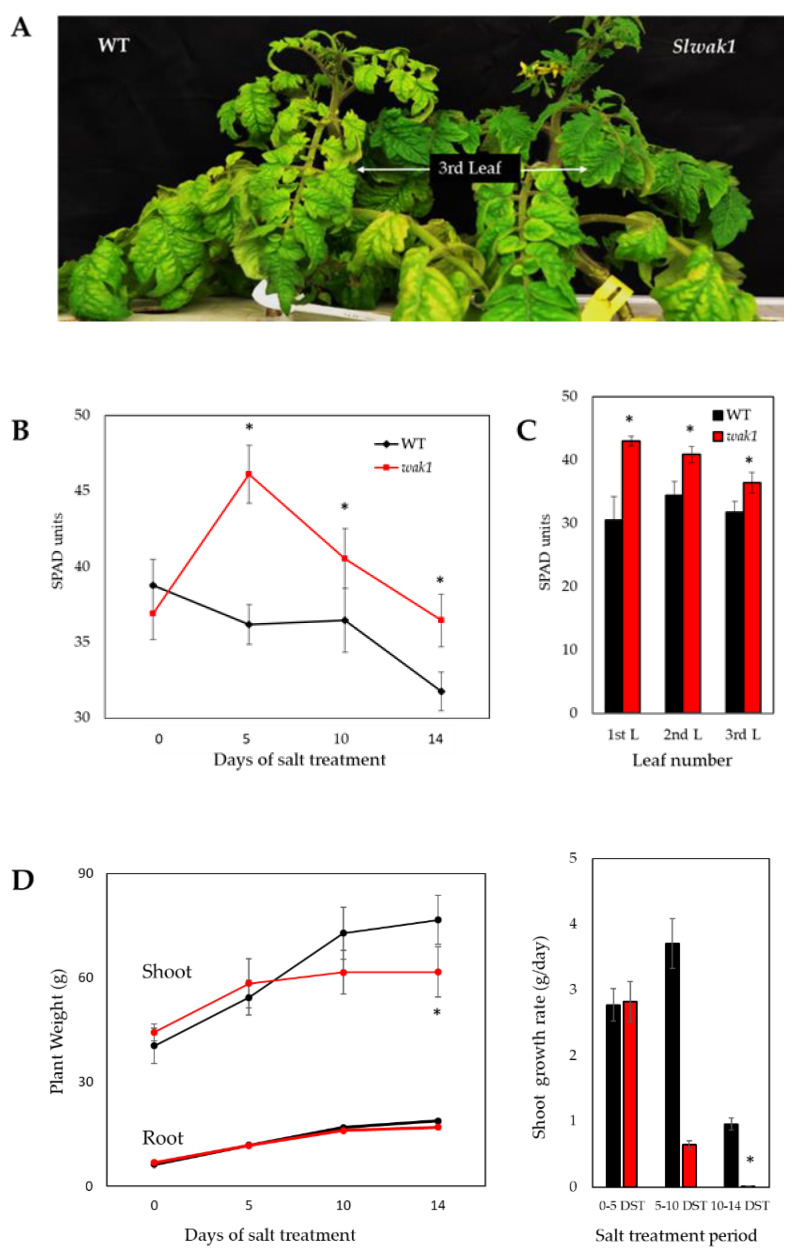
The null *Slwak1* mutant shows salt-sensitivity in spite of its lower leaf chlorosis than WT under salt stress. (**A**) Representative images of WT and *Slwak1* salt-treated plants (200 mM NaCl for 14 days). (**B**) Temporal evolution of the chlorophylls levels in the third developed leaf of WT and *Slwak1* plants. (**C**) Changes in the chlorophylls levels of the first three developed leaves (from the apex) at the end of the experiment. (**D**) Evolution of shoot and root biomass and shoot growth rate during salt treatment period. Data are expressed as mean ± SE (*n* = 5). Asterisks indicate significant differences between mean values of WT and *Slwak1* (Student’s *t*-test, *p <* 0.05).

**Figure 4:**
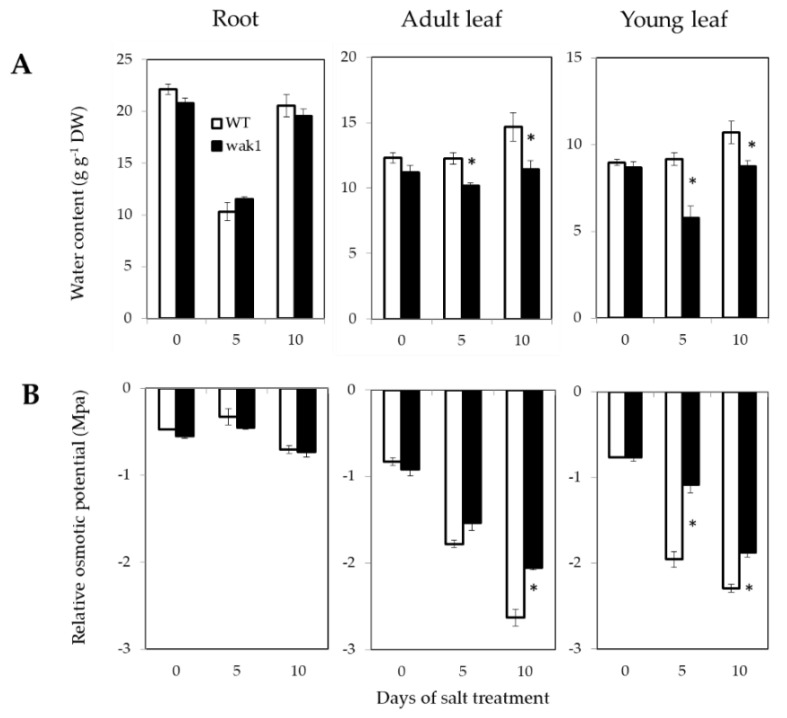
The *Slwak1* mutant is affected in osmotic homeostasis under salt stress. (**A**) Water contents and (**B**) osmotic potentials, relative to water content of hydrated cell, were analyzed in roots, adult and young leaves of WT and *Slwak1* plants after 0, 5 and 10 days of salt treatment (200 mM NaCl). Data are expressed as mean ± SE (*n* = 5). Asterisks indicate significant differences between mean values of WT and *Slwak1* at the same organ and culture condition (Student’s *t*-test, *p <* 0.05).

In order to confirm that the phenotype of *Slwak1* is in fact due to the *SlWAK1* mutant allele with a T-DNA insertion we have analyzed the phenotype of another tomato mutant of very different nature, where a single point mutation has been found in the coding sequence of this gene. This mutant belongs to a population generated in cv. TPAADASU by chemical mutagenesis applying ethyl methanesulfonate (EMS), a mutagenic agent causing SNPs in the DNA. This tomato EMS mutant line annotated as 12,007 was identified during the screening of the mutant population by means of TILLING (Targeted Induced Local Lesions IN Genomes) strategy [13]. This line presents a single SNP mutation in the coding sequence of Solyc09g014720 gene (*SlWAK1*) in position 1240 (C→T), giving rise to a premature stop codon and therefore a truncated protein of 413 residues, instead of the 799 residues of the wild type SlWAK1 protein. This truncated protein has lost the whole Ser/Thr kinase catalytic domain (STKc) (Figure S2A (Supplementary Materials)). Output data from HRM analysis shows the deviating curve that led to identification of 12,007 EMS mutant (Figure S2B (Supplementary Materials)). This mutant did not show any particular phenotype when grown in control conditions, without stress. The homozygous 12,007 EMS line was then grown in hydroponics culture and subjected to salt stress (200 mM NaCl), where azygous plants have been used as controls of the experiment. EMS line 12,007 exhibited remarkable lower leaf chlorosis than the azygous line after 7 and 10 days of salt treatment (DST) (Figure S2C (Supplementary Materials)), and a significantly higher chlorophylls content in 1st, 2nd and 3rd leaf after 10 DST (Figure S2D (Supplementary Materials)). Finally, 12,007 exhibited a significant reduction of shoot weight and leaf water content compared with azygous line after 10 DST, while no significant differences were found between both lines with respect to root weight and root water content (Figure S2E (Supplementary Materials)). These results regarding the phenotype of EMS mutant in salinity are analogous to those observed in *Slwak1* insertional mutant subjected to same salt stress conditions (Figure 2B,C, Figure 3A,C,D and Figure 4A), and they confirm that the phenotype of the latter is indeed due to the T-DNA insertion in *SlWAK1* gene.

WT and homozygous *Slwak1* mutant plants were grown in controlled conditions chamber without (control) and with salt stress applied at the 6th leaf stage. Firstly, the expression pattern of *SlWAK1* gene was analyzed by RT-qPCR in different organs of WT and *Slwak1* plants. In WT, expression of the target gene increased from apex to root, with very low levels in the youngest organs (apex, young leaf and upper stem) and the highest expression found in root (Figure 1E). The *Slwak1* mutant is a knockout insertional mutant as we did not find expression of the tagged gene, not only in the shoot but also in the root, in none of the three different experiments carried out.

The next step was to analyze the temporal expression of the *SlWAK1* gene in roots of WT plants submitted at 100 mM NaCl during the first 24 h and subsequently at 200 mM NaCl (Figure 1F). The *SlWAK1* expression was rapidly reduced during the first 24 h in 100 mM NaCl was applied. Four days (96 h) after that *SlWAK1* expression increased back to the initial level before salt stress was imposed, while during this period the NaCl level was increased to 200 mM. Such relatively rapid inhibition and posterior recovery of gene expression in salt stress conditions could be related to the fact that the gene product is involved in perception and signal transduction of external stimuli, in this case of pectin fragments generated from cell wall [8,9]. As the tagged gene is localized in a cluster surrounded by other WAK-coding genes (*SlWAK2* and *SlWAK3,* solyc09g014710 and solyc09g014730 respectively), we analyzed the expression of these flanking genes in roots of WT and *Slwak1* plants after 0, 48 and 120 h of salt treatments (Figure 1G). The expression of both genes showed comparable patterns in mutant and WT.

### 2.2. Phenotype and Physiological Responses of the Slwak1 Mutant to Salt Stress

The homozygous line of *Slwak1* mutant exhibited a phenotype of reduced leaf chlorosis and curliness compared with WT after 14 days of salt treatment (DST) (Figure 3A), as previously observed in the segregating population (Figure 2B,C). The temporal evolution of chlorophylls measurement in the third developed leaf of WT and *Slwak1* plants, confirmed that the deeper green color in *Slwak1* compared with WT leaves is associated with higher chlorophylls content under salt stress (Figure 3B). This physiological trait was maintained in the youngest part of the shoot, as determined by high chlorophylls levels in the youngest three developed leaves at the end of experiment (Figure 3C). Although higher leaf chlorophyll content is generally associated with higher salt tolerance [5], the opposite response was observed in the *Slwak1* mutant. *Slwak1* mutant showed a similar plant growth to WT during the first 5 DST, but after that its shoot growth rate became much lower than that of WT, and significant differences between shoot growth of WT and *Slwak1* plants were measured at 14 DST. However, root growth was similar in WT and the *Slwak1* mutant (Figure 3D).

Because of the lower water potential due to the salt concentration outside the roots, the first effect induced by salinity is osmotic stress. In order to determine whether the *SlWAK1* disruption alters the osmotic homeostasis, water content was analyzed in roots, adult and in young leaves of WT and *Slwak1* during the first 10 DST (Figure 4A). The water content was similar in WT and *Slwak1* plants under control conditions but not under salt stress. A significant reduction in water content was observed in adult leaves and, especially in young leaves of *Slwak1* mutant after 5 and 10 DST, which indicated a clear inability of the mutant to avoid dehydration in the shoot, while the water content was similar in roots of WT and *Slwak1*.

Considering that leaf dehydration induced by salinity may be due to lower capacity to reduce osmotic potential (ᴪ_s_) by means of solutes accumulation, we analyzed ᴪ_s_ in roots, adult leaves and young leaves. Bearing in mind that the ᴪ_s_ values reflect not only the reduction due to the active accumulation of solutes but also the solute concentration due to cell dehydration, we calculated the ᴪ_s_ values relatives to the water content of hydrated cell (Figure 4B). These values showed that the significant differences between adult leaves of WT and *Slwak1* aroused after 10 DST, and in young leaves after 5 and 10 DST. Reduction of ᴪ_s_ relative values was remarkably lower in *Slwak1* compared with WT in both types of leaf in those time periods of salt stress. Leaf dehydration may be also due to higher water loss through the leaf. Therefore, to estimate the water loss through the leaf, the weight loss of detached leaflets from the fourth leaf of plants grown in control was measured. This parameter was analyzed at short term (during 9 h) and at long term (from 1 to 8 days) (Figure S3 (Supplementary Materials)). The values of leaflet weight loss were identical in WT and *Slwak1*, indicating that the *SlWAK1* disruption did not affected the water loss through the leaves. Taken together, the higher salt sensitivity of the *Slwak1* mutant seems to be due to its lower ability to recover osmotic homeostasis.

**Figure 5:**
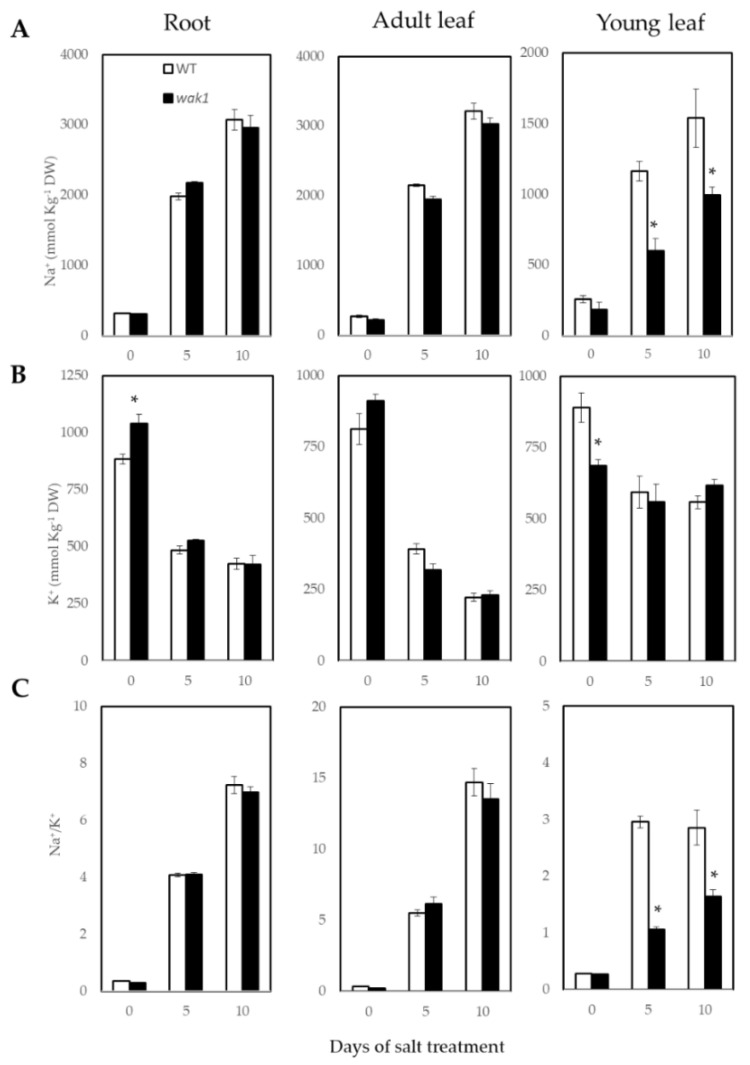
The *Slwak1* mutant is tolerant to Na^+^ homeostasis under salt stress. (**A**) Na^+^ and (**B**) K^+^ concentrations, and (**C**) Na^+^/K^+^ ratio in roots, adult and young leaves of WT and *Slwak1* plants after 0, 5 and 10 days of salt treatment (200 mM NaCl). Data are expressed as mean ± SE (*n* = 5). Asterisks indicate significant differences between mean values of WT and *Slwak1* at the same organ (Student’s *t*-test, *p* < 0.05).

Under salt stress, maintenance of chlorophyll content in young leaves may be a consequence of the tolerance to the Na^+^ toxicity, as tomato plants accumulate Na^+^ mainly in roots and adult leaves to prevent Na^+^ from reaching toxic levels in young developing tissues. Analysis of Na^+^ concentration during the first 10 DST, demonstrated that the Na^+^ content was similar in WT and *Slwak1* mutant roots and adult leaves, while in young *Slwak1* leaves the Na^+^ level was significantly lower after 5 and 10 DST (Figure 5A).

Regarding K^+^ content, the only differences between WT and *Slwak1* mutant were found under control conditions, with *Slwak1* showing higher content in root and lower in young leaf, compared with WT, whereas these differences disappeared under stress (Figure 5B). A trait generally associated to salt stress tolerance is the Na^+^/K^+^ ratio. In our study, the Na^+^/K^+^ ratio significantly decreased in young leaves of *Slwak1* salt-treated plants, which was due to Na^+^ content reduction in this organ, whereas no significant changes were found in the other plant parts analyzed (Figure 5C). These results suggest that *Slwak1* mutant is more tolerant to ionic stress induced by salinity. In order to know whether the main Na^+^ transporters involved in the long-distance transport from root to shoot were affected by the *SlWAK1* mutation, we analyzed the level of transcripts of *SlSOS1* and *SlHKT1.2* in roots (organ where this gene is mostly expressed) from 0 to 5 days of 200 mM NaCl treatment (Table S1 (Supplementary Materials)). No significant differences between WT and *Slwak1* mutant were found in expression of these Na^+^ transporters, which indicates that the lower Na^+^ accumulation in young leaves of *Slwak1* is not associated with a reduction of Na^+^ transport from the roots.

### 2.3. Changes in the Metabolite Profiles of Source Leaf and Sink Root of Slwak1 Mutant Under Salt Stress

**Figure 6:**
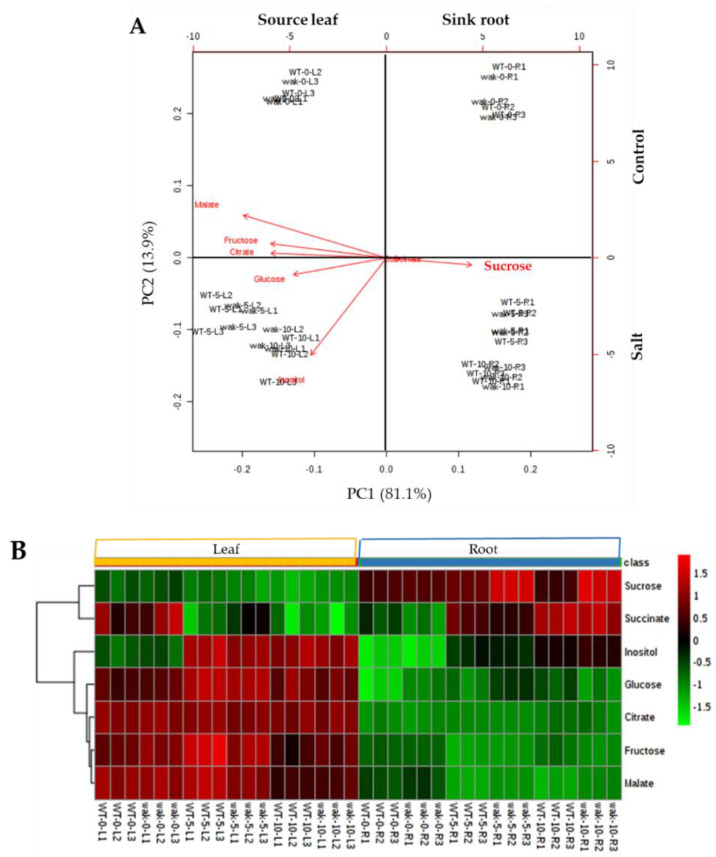
Changes in the metabolite profiles induced by the disruption of *SlWAK1* in source leaf and sink root. Plants of WT and *Slwak1* were analyzed after 0, 5 and 10 days of salt treatment (200 mM NaCl). (**A**) Non-supervised principal component analysis (PCA) and (**B**) heatmap analysis representing the major sources of variability. Color scale represents the variation in the relative concentration of compounds, from high (red) to low (green) contents.

Until now we have analyzed changes in the transport of water and inorganic solutes from roots up to the shoots occurring via xylem, but the phloem is responsible for the transport of organic solutes from source tissues to sink tissues. Considering that the main source is the adult leaf and the main sink in tomato before fructification is root, we studied the changes of the main organic solutes (sugars and organic acids) induced by salinity in the source leaves and root sinks of WT and *Slwak1* mutant. Firstly, we performed PCA for leaf source and root sink together. PC1, which explains 81% of total variance, reflected the difference of metabolite profiles between both organs and PC2 (explaining by 13.9%) revealed the differences between control and salt stress, but differences between WT and *Slwak1* were not clearly showed (Figure 6A).

**Figure 7:**
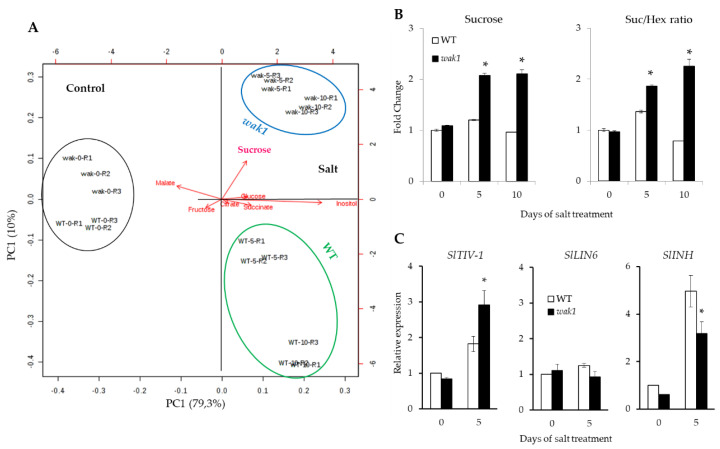
The disruption of *SlWAK1* induces a high sucrose accumulation in root under salt stress. (**A**) PCA-biplot in roots of WT and *Slwak1* plants grown at 0, 5 and 10 days of salt treatment (200 mM NaCl). Under salt stress, *Slwak1* mutant is clearly separated from WT due mainly to sucrose contribution. (**B**) Fold changes of sucrose and sucrose/hexoses ratio in roots of WT and *Slwak1* plants grown at 0, 5 and 10 days of salt treatment (200 mM NaCl). (**C**) Expression levels of key genes involved in sucrose cleavage into hexoses, the vacuolar invertase *SlTIV-1*, the cell wall invertase *SlLIN6*, and the invertase inhibitor *SlINH*, in roots of WT and *Slwak1* plants grown at 0 and 5 days of salt treatment (200 mM NaCl). Data are expressed as mean ± SE (*n* = 5). Asterisks indicate significant differences between mean values of WT and *Slwak1* at the same organ (Student’s *t*-test, *p* < 0.05).

Important differences were found between the metabolic profiles of leaf source and sink root as observed in the heatmap representation, and also some differences were detected for treatment time within each plant organ for succinate and inositol (Figure 6B). Interestingly, the PCA-biplot showed that sucrose greatly contributed to separate the samples of both organs by the PC1, with the higher positive coefficient being found for sucrose in root (Figure 6A). The heatmap representation clearly indicated that sucrose responses to salt were more intense in *Slwak1* mutant than WT, in line with PCA observations (Figure 6B). Next, we analyzed separately source leaf and sink root data in order to define whether there were differences in the metabolite profiles of WT and *Slwak1* organs. For source leaf, the PCA-biplot did not reveal separation between WT and *Slwak1* taken under control conditions; it did reveal separation between salt stress conditions (Figure S4 (Supplementary Materials)). This indicates that metabolite composition of the leaf source is only modified by the salt stress condition, but not by disruption of *SlWAK1* gene. In root, however, WT formed one group different from that of *Slwak1* under salt stress, which was not observed between control samples (Figure 7A).

Therefore, *Slwak1* mutant triggers a metabolic response after applying salt stress, which seems to be maintained by increasing the salt treatment period, as observed by differences found between 5 and 10 DST in the PCA-biplot. Moreover, fold changes of sucrose and sucrose/hexoses ratio show that the sucrose accumulation in the mutant root is similar between 5 and 10 DST, while the sucrose/hexoses ratio even increases slightly with the treatment time (Figure 7B). A hypothesis that would explain this observed high sucrose accumulation found in *Slwak1* root could be the reduced ability of the mutant to hydrolyze sucrose in hexoses. To test this hypothesis, we analyzed at 0 and 5 DST the expression levels of key genes involved in sucrose cleavage into hexoses: vacuolar invertase*SlTIV-1*, cell wall invertase *SlLIN6*, and invertase inhibitor *SlINH* (Figure 7C). No changes were observed in *SlLIN6* expression of WT and mutant roots, either in control or salt stress. However, expression of *SlTIV-1* was higher in *Slwak1* than WT under salt stress while the opposite response was found for *SlINH*, which supports the hypothesis that higher sucrose accumulation in *Slwak1* is inducing higher expression of vacuolar invertase.

### 2.4. The Slwak1 Mutant Exhibits a Higher Yield Penalty than WT at Long-Term Salt Stress

The main sinks change through the plant life cycle, being root the main sink organ before fructification, while fruit is the main sink in adult plants. In order to know whether salt stress continue to negatively affect *Slwak1* growth to long term, we measured fruit yield of WT and *Slwak1* plants grown without salt (control) and with salt stress (100 mM NaCl). In a first experiment, we observed that fruit yield was significantly lowered in *Slwak1* than WT plants grown under salt stress, which did not occur in control conditions (Figure S5 (Supplementary Materials)).

**Figure 8:**
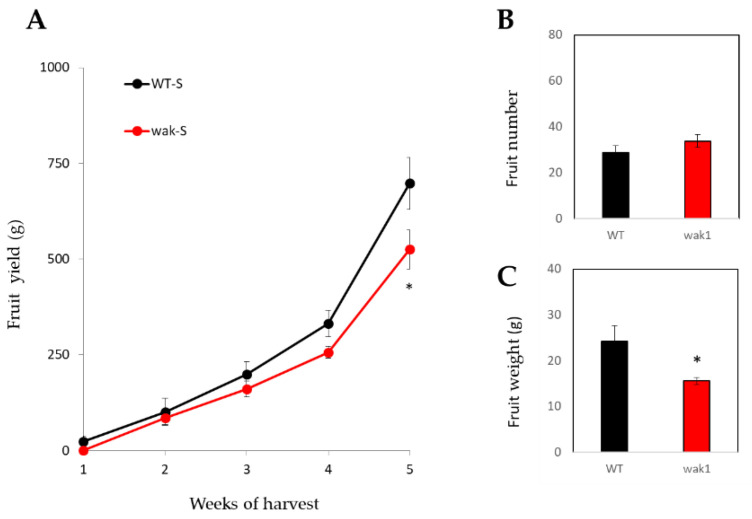
The null *Slwak1* mutant shows salt sensitivity to long term (**A**) WT and *Slwak1* mutant plants were subjected to salt stress (100 mM NaCl), and red fruits were collected for 5 weeks, determining fruit number (**B**) and (**C**) fruit weight. Values are expressed as mean ± SE (*n* = 8). Asterisks indicate significant differences between mean values of WT and *Slwak1* (Student’s *t*-test, *p* < 0.05).

Subsequently, we carried out another experiment with WT and *Slwak1* plants with the objective of testing how the *SlWAK1* mutation was able to affect fruit development, as this is a process mainly related with the transport of photoassimilates (sucrose) from source leaf to sink (fruit). The accumulated production supported the data of the previous assay, since fruit yield was significantly lower in *Slwak1* than WT at the end of the harvest period (Figure 8A). This was due to the reduced fruit weight (Figure 8C) not to a reduced fruit number (Figure 8B) as expected taking into account that fruit expansion is strongly related to availability of photoassimilates.

## 3. Discussion

Up to our knowledge, the role of *SlWAK1* has only been studied in the biotic stress tolerance of tomato [10,11], while there are no reports on the role of this gene in the tomato response to abiotic stress. One of the problems in order to elucidate the role of *WAK* genes might be their functional redundancy, as recently pointed out by Zhang et al. [11]. To solve these limitations, these authors developed two CRISPR/Cas9-mediated *Slwak1* mutants in tomato, using them to further advance in the knowledge on the role of *SlWAK1* in biotic stress tolerance. Here, we have identified a knockout mutant for *SlWAK1* in roots, the plant organ where *SlWAK1* is mainly expressed (Figure 1E). However, expression of *SlWAK2* and *SlWAK3* flanking in roots is not null; in that case the mutant could be lethal (Figure 1G). In *A. thaliana*, transgenic plants constitutively expressing *WAK1* or *WAK2* antisense transcripts, which silence the whole *WAK* family, were not obtained, suggesting that loss of the *WAK* function determines lethality [14]

WT and *Slwak1* mutant plants were very similar when grown under control conditions (Figure 2A). It is possible that under these conditions *SlWAK1* function is redundant and masked by the presence of *SlWAK2* and *SlWAK3*. The fact that *Slwak1* mutant exhibits a specific response to application of salt stress is very interesting and suggests a specific function in the salt tolerance mechanism. This specific function of *SlWAK1* with respect to *SlWAK2* and *SlWAK3* may be related to specific regulation of its activity resulting from differential post-translational modifications. In any case this is an issue that merits further investigation. Under salt stress, *Slwak1* mutant exhibited a phenotype of reduced leaf chlorosis with respect to WT (Figure 2B,C, and Figure 3A), which was associated to higher chlorophylls levels in leaves under salt stress (Figure 3B,C), a trait associated to salt tolerance [6]. However, contrarily to what we expected, *Slwak1* mutant is salt-sensitive on the basis of vegetative growth, although the negative effect of salt stress on plant growth of *Slwak1* mutant was not observed during the first days of salt stress but to mid-term (Figure 3D) and, especially, to long-term on the basis of fruit yield (Figure 8A and Figure S5 (Supplementary Materials)). In accordance with our observations, also in the halophyte *Nitraria sphaerocarpa*, expression of a *WAK* gene (*WAK4)* was only increased after a long exposure to salt amplifying the effects of Na^+^ [15]. The abiotic stress tolerance has been even associated to down-regulation of *WAKs*, as observed in the resurrection plant *Craterostigma plantagineum*, a halophyte very tolerant to dehydration [16]. Taken together, our results strongly suggest that *SlWAK1* is involved in tolerance to long-term salt stress in tomato.

### 3.1. Slwak1 Mutant Does Not Recover Osmotic Homeostasis But It Is Tolerant to Na^+^ Homeostasis

Salinity tolerance depends on both, osmotic tolerance to avoid the osmotic effect of the salt outside the roots and ionic tolerance to avoid the toxic effect of the salt within the plant. It is noteworthy the different response mechanisms of WT and mutant to osmotic homeostasis, as water contents in *Slwak1* leaves were reduced throughout the treatment period, while in WT leaves were maintained and even increased (Figure 4A). These results indicate that *Slwak1* is unable to recover water transport from the root to the shoot under salt stress. This fact is related to its lower capacity to reduce osmotic potential relative to water content of hydrated cell, in adult and, especially, young leaves, and it is not related to water loss through the leaves (Figure 4B and Figure S3 (Supplementary Materials)). Recently, Zhang et al. [11] did not observe differences in stomata number and conductance between WT and *Slwak1* tomato mutant generated by CRISPR/Cas9, suggesting that *SlWAK1* does not play a role in leaf transpiration. In sum, the *Slwak1* mutant does not recover the osmotic homeostasis under salt stress and, therefore, growth reduction induced by salinity in *Slwak1* mutant must be partially associated to osmotic homeostasis. In this sense, several studies demonstrate that osmotic homeostasis can be so important or even more important than ion homeostasis in salt tolerance of tomato [6,17].

Initially, we selected the *Slwak1* mutant by its supposed salt tolerance due to the fact that its leaves remain greener under salt stress than WT leaves (Figure 2B,C and Figure 3A,B), a trait related to Na^+^ toxicity. In fact, *Slwak1* mutant seems more tolerant to ion homeostasis based on lower Na^+^ content in young leaves when compared with WT leaves (Figure 5A). This assumption is also supported by the lower values of Na^+^/K^+^ ratio in young leaves (Figure 5C), since this ratio is considered a physiological trait associated to salt tolerance in tomato [18]. One question to discern here was whether knockout of *SlWAK1* altered the gene expression of the main Na^+^ transporters. The similar expression levels in *Slwak1* and WT roots of the two main genes involved in Na^+^ transport from root to shoot, *SlSOS1* and *SlHKT1.2* [19,20] (Table S1 (Supplementary Materials)), demonstrates that tolerance of *Slwak1* mutant to ion homeostasis is not associated to a differential expression of Na^+^ transporters but rather to its low capacity to transport water and solutes from root to shoot (Figure 4A,B). These results show that *Slwak1* mutant is tolerant to Na^+^ homeostasis but not to osmotic homeostasis and, therefore, the higher salt sensitivity of *Slwak1* is a consequence of the prevalent effect of osmotic stress throughout its life cycle.

### 3.2. Sucrose Distribution between Source Leaf and Sink Root Is Altered in Slwak1 Mutant Under Salt Stress

Plant growth is dependent on the source-sink relationship, which is influenced by salt stress [21]. The main sink organ prior to fructification is the root and its metabolic profile is a key aspect to comprehend the source-sink balance. Interestingly, Rivero et al. [22] observed that the metabolic profile of tomato roots was drastically altered when plants were subjected to salinity, while this did not occur when plants were subjected to drought stress. This observation reveals the different strategies tomato plants adopt when confronted with each type of abiotic stress, even if they share the osmotic effect. During its life cycle, the plant undergoes changes in the metabolism of both source and sink as well as in the degree of competition among various sinks [23]. Keeping in mind that the main source organ is adult leaf and the main sink organ in tomato before fructification is root, we analyzed the changes in the main organic solutes, sugars and organic acids, induced by disruption of *SlWAK1* in adult leaf (source) and root (sink) plants. The PCA analysis reflected that sucrose contributed greatly to separate the samples of both organs, leaf source and root sink, being the high sucrose accumulation induced by salt stress in *Slwak1* roots the main metabolic change induced by disruption of *SlWAK1* (Figure 6A and Figure 7B). In spite of the relatively high degree of divergence observed between *WAK* genes in Arabidopsis and tomato, it is interesting to remark that the main metabolic alteration found in the Arabidopsis *wak2* mutant was that of sucrose metabolism in roots [24], concluding that *AtWAK2* regulates expression of the vacuolar invertase *AtINV1*.

Since sucrose is the main way of transporting photoassimilates from source to sink organs [23], the high root sucrose accumulation in *Slwak1* may be due to either higher transport towards root or lower sucrose hydrolysis in hexoses within the root. When we analyzed in roots the expression of key genes involved in sucrose cleavage, we observed that the expression of vacuolar invertase *SlTIV-1* increased in *Slwak1* root compared with WT under salt stress, while the opposite response was found for the invertase inhibitor *SlINH* (Figure 7C). These results support a higher sucrose transport to *Slwak1* roots, probably to uphold the low capacity of the mutant to maintain osmotic homeostasis after application of salt stress; moreover, it is necessary to take into account that osmotic potential reduction is lower if sucrose accumulates rather than hexoses, as it is occurring in *Slwak1* roots.

Increases in tomato productivity will depend on a higher understanding on how sources or sinks limit plant growth and how these change during the life cycle [25]. Despite the numerous studies on source-sink ratios there is still a controversy on which processes mainly control plant growth and hence final crop yield [26]. Furthermore, the high sucrose transport towards roots may go at the cost of other sink organs [21]. Since fruit is the main sink in adult plants, we studied the agronomic response of *Slwak1* to determine whether the negative effect induced by salinity in plant growth was maintained along the life cycle. In fact, fruit yield was significantly reduced by salinity in *Slwak1* compared with WT, with yield reduction being only due to lower fruit weight (Figure 8A,C and Figure S5 (Supplementary Materials)). This is in agreement with the fact that fruit development is mainly related to transport of photoassimilates via phloem, mainly sucrose, which seems to be the main process affected in *Slwak1*.

In summary, up to our knowledge, the results presented here are the first ones showing the role of *SlWAK1* in the tomato plant response to salt stress. It has been demonstrated that the disruption of this gene affects salt tolerance along the life cycle of tomato, mainly due to its inability to re-establish osmotic homeostasis. The most important metabolic alteration in *Slwak1* mutant is its increased sucrose accumulation in roots, which alters the source-sink relationship. Future studies are planned to elucidate how the metabolic flux drives towards sucrose accumulation in roots as well as the mechanisms that underlie the assimilates partitioning between source and sink organs.

## 4. Materials and Methods

### 4.1. Identification of Tomato Slwak1 Mutant and Gene Cloning

The tomato (*S. lycopersicum* L.) cv Moneymaker was used to generate a collection of insertional mutants using T-DNA by means of the enhancer trap vector pD991 [12]. Screening for salt tolerance was performed as previously described [27]. At least 12 plants coming from segregating populations (T_2_) were grown in hydroponic culture, using half-strength Hoagland solution [28]. Salt treatment (200 mM NaCl) was initiated when plants had developed two true leaves. A tomato mutant was selected by its supposedly salt tolerance as consequence of the T_2_ segregation for leaf chlorosis.

The number of T-DNA copies was determined by Southern blot hybridization experiments. Genomic DNA was isolated from young leaves as described by Dellaporta et al. [29]. Ten μg of genomic DNA was digested with *EcoR*I and *Hind*III endonucleases, separated by gel-electrophoresis in 0.8% agarose gel, and blotted onto Hybond N^+^ membranes (GE Healthcare) as described by Brown [30]. Hybridization was performed with a chimeric probe, fusing the complete coding sequence of the *NptII* gene to 811 pb of the coding sequence from the endogenous tomato *FALSIFLORA*(*FA*) gene, which was employed as hybridization positive control [31]. The genomic flanking sequences surrounding the T-DNA insert were isolated by anchor-PCR according to the procedure described in Pérez-Martín et al. [12]. The sequences of primers used in the anchor-PCR are listed in Table S2 (Supplementary Materials).

Genotyping of the segregating T_2_ progeny was performed by PCR using (i) the specific genomic forward (WAK1-F) and reverse (WAK1-R) primers to amplify the WT allele (without T-DNA insertion) and (ii) one specific genomic primer (WAK1-F) and the specific T-DNA border primer (ARB-2) to amplify the mutant allele (carrying the T-DNA insertion). Primers were designed based on sequence information available from SGN Database (http://solgenomics.net/). The same progeny was analyzed by PCR for the presence of the marker gene *NptII*. The primers sequences for genotyping and for amplification of *NptII* are listed in Table S2 (Supplementary Materials). For PCR amplification DNA extraction was performed with Plant DNAzol Reagent (Invitrogen, Carlsbad, CA), following the manufacturer instructions. Amplification by PCR was performed in a 30-μL volume using 25 ng of total DNA, 50 ng of each primer, 0.25 mM dNTPs, 2.5 mM MgCl_2_, and 1 U of RED-Taq DNA polymerase (SIGMA-Aldrich) in 1× Taq buffer. DNA was amplified under the following thermal cycling conditions: 94 °C for 5 min, followed by 35 cycles at 94 °C for 30 s, 60 °C for 30 s, and 72 °C for 2 min, and a final extension of 5 min at 72 °C. The PCR products were analyzed in 1% agarose gels in SB buffer (10 mM sodium boric acid, [32]) and visualized with ethidium bromide.

### 4.2. Identification of the Tomato EMS Mutant Affected in SlWAK1

Tomato cv. TPAADASU, a highly homozygous inbred parental line, was subjected to ethyl methanesulfonate (EMS) treatment to generate an EMS mutant population, characterized by presenting point mutations (SNPs). This population was screened for SNPs making use of the TILLING (Targeted Induced Local Lesions IN Genomes) strategy [13]. A high throughput seed DNA extraction was set up and screening of the mutant population was performed by means of High-Resolution Melt curve analysis (HRM) [13]. To identify EMS mutants with SNPs in Solyc09g014720 gene (*SlWAK1*) coding sequence a set of three primers were designed for the PCR step prior to application of HRM (Table S2 (Supplementary Materials)).

For designing these primers, the *SlWAK1* region between amino acidic residues 391 and 739 was considered since this region contained most of the sequence which when mutated gives rise to deleterious mutations. This region contains 138 (including Stop codons) chances for a mutation to be deleterious. Next to this we have 23 times the chance to find a mutation that causes a stop codon.

The generation of the EMS mutant population and its screening by TILLING was performed by Nunhems Netherlands BV company (Haelen, The Netherlands).

The 12,007 EMS line containing a single SNP in Solyc09g014720 sequence in homozygosis, giving rise to a Stop codon, was grown in hydroponics culture and subjected to salt stress (200 mM NaCl) for 10 days, as described elsewhere. Azygous plants resulting from the segregation of 12,007 line, where the SNP is absent, have been used as controls of the experiment.

### 4.3. Gene Expression Analyses

Expression analysis of WAK-coding genes *SlWAK1* (Solyc09g014720), *SlWAK2* (Solyc09g014710) and *SlWAK3* (Solyc09g014730); those genes coding for ion-transporters involved in Na^+^ homeostasis *SlSOS1* (Solyc01g005020) and *SlHKT1.2* (Solyc07g014680), and genes involved in sucrose cleavage vacuolar invertase *SlTIV-1* (Solyc03g083910), cell wall invertase *SlLIN6* (Solyc10g083290), and invertase inhibitor *SlINH* (Solyc12g099200), were analyzed in roots of WT and *Slwak1* plants, and *SlWAK1* in different parts of the plants, by quantitative real-time PCR (RT-qPCR) at 0 DST and during 5 days of salt treatment as previously described [5]. Plant samples were immediately frozen in liquid nitrogen and stored at −78 °C until gene expression analysis. Total RNA was isolated from apex, leaves, and stem of WT plants in control (without NaCl) conditions, and roots of NaCl-treated and non-treated WT and *Slwak1* plants using the RNeasy Plant MiniKit (Qiagen), and then treated with RNase free DNase (Qiagen) to remove contaminating DNA, according to the manufacturer’s protocols. RNA extracts were finally re-suspended in RNase free water and immediately aliquoted to prevent RNA degradation. The expression level of targeted genes was assessed by real-time PCR using gene-specific primers listed in Table S2 (Supplementary Materials). Real-time RT-qPCR was performed using Rotor-Gene™ SYBR^®^ Green (Qiagen) in a Rotor-Gene 3000 (Corbett Life Science). First-strand cDNAs were synthesized from 1 µg of total RNA using iScript™ Reverse Transcriptase kit (BIO-RAD) according to the manufacturer’s protocol. Serial dilutions of cDNA were used to make a standard curve to optimize amplification efficiency. All reactions were performed in triplicate. Melting curves of the reaction products were generated and fluorescence data were collected at a temperature above the melting temperature of non-specific products. Relative expression data were calculated from the difference in threshold cycle (ΔCt) between the studied genes and DNA amplified by primers specific (Table S2 (Supplementary Materials)) for the tomato elongation factor 1𝛼 (*EF1𝛼*, Solyc06g005060) [20]. A Ct of around 21 cycles in WT (Moneymaker) roots in control conditions was the value for *SlWAK1*. In WT leaves in control conditions the expression was very low (around 29 cycle), a result supported by other very recent reports [11]. The number of RT-qPCR cycles performed was 35. The expression level was calculated using 2^−ΔΔCt^ method [33], considering the expression level from root in control conditions as the calibrator sample.

### 4.4. Gene and Protein Sequence Bioinformatics Analysis

The cloned sequences were compared with SGN database (https://solgenomics.net) to localize the T-DNA insertion site within the tomato genome. pI and MW predicted values of the translated *SlWAK1* sequence was found out using the ExPASy tool (https://web.expasy.org/compute_pi/). Multiple sequence alignment was conducted with ClustalW and the phylogenetic tree was constructed using rooted tree with branch length (UPGMA), making use of GenomeNet bioinformatics tools (https://www.genome.jp/tools-bin/clustalw). The homologous sequences of SlWAK1 were retrieved from NCBI and SGN databases or the scientific literature: wheat from [34], *C. plantagineum* from [16], *Oriza sativa* from [35], *A. thaliana* from [36], those from Solanaceae species other than domesticated tomato (*S. pennellii*, potato and pepper) from GenBank after submitting SlWAK1 sequence to BLASTP (https://blast.ncbi.nlm.nih.gov/) [37], and other tomato homologous gene sequences were obtained from the SGN (https://solgenomics.net) using the predicted SlWAK1 protein sequence (Solyc09g014720.1.1). Protein domains were identified using the NCBI Conserved Domain (CD)-Search tool (http://www.necbi.nlm.nih.gov/Structure/cdd/wrpsb.cgi [38]). The N-terminal signal peptide and transmembrane domain were predicted using SignalP 4.1 Server (http://www.cbs.dtu.dk/services/SignalP/ [39]) and TMHMM Server V.2.0 (http://www.cbs.dtu.dk/services/TMHMM/), respectively.

### 4.5. Characterization of Slwak1 Mutant

Tomato wild type (WT, cv Moneymaker) and T_3_ homozygous line for the *Slwak1* mutation were used for the phenotypic and physiological characterization of the *Slwak1* mutant. Plant culture was carried out in a controlled growth chamber as previously described [2]. The hydroponic system consisted in tanks of 50 L capacity filled with half-strength (½) Hoagland nutritive solution [27], which was continuously aerated by means of a compressor (Puska N-150-150, with a 115 L min^−1^ flow, 10 Kg cm^−2^ maximum pressure and 50 L capacity). Hydroponic solution was controlled by monitoring pH and electrical conductivity (EC), renewing the solution at least once per week. When plants reached a stage of three-four fully-developed leaves, salt treatment of 200 mM NaCl during 14 days was applied. To avoid osmotic shock salt was firstly applied at 100 mM NaCl during 24 h. Five plants of each genotype were harvested after 0, 5, 10 and 14 days of salt treatment (DST). Two independent salinity experiments were performed following the same experimental design.

In each selected time of salt treatment, shoot and root biomass accumulation (g fresh weight) was calculated by weighing individual plants. Chlorophylls content was determined by means of a leaf chlorophyll meter (SPAD-502, Minolta, Kyoto, Japan). The Soil Plant Analysis Development (SPAD) units that the equipment displays are correlated with the plant chlorophylls content [40]. Measures were collected in three different leaflets of a tagged leaf and average values calculated.

In each harvest, fresh material was rinsed with deionized water and carefully blotted with tissue paper. Selected organs of the plants were sampled, as shown in Results. Part of the material was weighed for fresh weight (FW) measurement, oven dried for 48 h at 80 °C and weighed to determine dry weight (DW) and then the water content was found out (FW-DW/DW, expressed as mL H_2_O g^−1^ DW). Another part of the material was placed in 5 mL-pipette tips containing glass wool at the tip, immediately frozen in liquid nitrogen and stored at −80 °C until analysis. Subsequently, the sap was extracted from the thawed material by centrifugation and used for physiological analysis. For molecular analysis, the selected plant material was immediately frozen in liquid nitrogen and stored at −80 °C.

Osmotic potential (ψ_s_) was determined by measuring osmolality in the sap extracts by the freezing point depression method using an osmometer (Roebling DR 02, Berlin, Germany). Osmolalities (mOsm Kg^−1^) were converted to osmotic potential (1 mOsm = −2.408 kPa). Leaf water loss rate was determined in detached leaflets from the fourth leaf of *Slwak1* and WT adult plants, placed on open-lid Petri dishes, immediately weighed and incubated for 8 days. The decrease in FW was monitored along the assayed time and results expressed as percentage of weight loss relative to initial FW.

The concentration of Na^+^ and K^+^ ions in the selected plant material was determined in oven-dried material, milled to powder and digested in a HNO_3_:HClO_4_ (2:1 v/v) solution. The ions were analyzed by inductively coupled plasma optical emission spectrometry (ICP-OES) at the Ionomics platform of CEBAS-CSIC (Murcia, Spain).

Long-term salt stress assays with WT and *Slwak1* plants were performed in a polyethylene greenhouse where plants were grown on coconut peat, using a drip irrigation system, with 3 L h^−1^ drippers. The fertirrigation solution (Hoagland solution) was prepared in 2000 L tanks with local irrigation water (EC = 0.9 dS m^−1^) and salt treatment consisted of adding 100 mM NaCl to the tank [2]. The salt treatments were applied from 15 days after transplantation until the end of the experiment. Ripe fruits were collected weekly for approx. 2 months, corresponding approx. to the 40–80 days period of salt treatment and fruit number and weight were recorded.

### 4.6. Analysis of Sugars and Organic Acids

Sucrose, hexoses (fructose and glucose), inositol and organic acids (citrate, malate and succinate) were determined by a HPLC-UV/RID equipment (Shimadzu, Kyoto, Japan), using a thermostated ion-exchange column (ION-300, Teknochroma). A diluted solution of the sap extracts obtained to measure osmotic potential were used for this analysis that was performed according to Sanchez-Bel et al. [41].

### 4.7. Statistical Analysis

Data were statistically analyzed using the SPSS 13.0 software package by one-way analysis of variance (ANOVA) using the genotype as factor, and least significant difference (LSD) or Student’s *t*-test considering a confidence level of 0.05. Significant differences among mean values were denoted by different lower-case letters or asterisks. All data are expressed as mean ± SE (*n* = sample size).

For multivariate analysis, PCA-biplot and heatmap were performed on these data matrixes and used to ascertain the overall variability both among organs (source leaf and sink root), genotypes (WT and *Slwak1* mutant) and treatments (control and salt), and between genotypes and treatments per organ. Multivariate analysis was produced using the Metaboanalyst 4.0 server [42]. Firstly, raw data were normalized by median, processed using generalized log transformation (log 2) and then mean-centered and divided by the square root of deviation of each variable (Pareto scaling). The univariate analysis of fold change was also performed using the Metaboanalyst 4.0 server.

## Supplementary Materials

Supplementary Materials can be found at https://www.mdpi.com/1422-0067/21/17/6308/s1.
