# Supplementary material for: The Salt Sensitivity Induced by Disruption of Cell Wall-Associated Kinase 1 (SlWAK1) Tomato Gene Is Linked to Altered Osmotic and Metabolic Homeostasis
Source: Int J Mol Sci. 2020 Aug 31;21(17):6308. doi: 10.3390/ijms21176308 (PMC7503591; doi:10.3390/ijms21176308)
Supplement: Supplementary file 1 [file ijms-21-06308-s001.pdf]

```
Solyc09g014740.    1 -MQLKQIALFF-SFTCSVL-------TILTSISA-----------HPTADSPVSPTTITKAPNIAKPGCPKQCGNVTIPY
Solyc09g014730.    1 MMKQCQLLLLH----FFLIFLTLANA-----------------------QTTTNTTITKGANITKPGCPKKCGNITIPY
Solyc09g014720.    1 -MQHYQVALFSFQLPCFMLILTLATAQIIPSNTTSPPTNSTSPPTNATAPAPSPTNTITKAANITKPGCPKQCGNVTVPY
Solyc09g014710.    1 -MQLRQLPF----FISFMLILTLDTAQIIPSNTTPPPTNTTSP-----PITAATTTITKAVIITKPGCPKKCGNLTVPY
consensus          1 .*...*.    ...  ....... ........... ..   .. ....... .   ..****.   *.******.***.*.**

Solyc09g014740.   61 PFGI--GSDCAMDSSFEINCTAGS--SATLYRSNIKIYDISDSEIRVSNMLYQRCYSETGQLLVPDNPTWLRFGKLTPYS
Solyc09g014730.   53 PFGIGLGLGCAFDSSFEIGCNKST-GTPTLYASNIKVYDISDAETRVFNFIAETCYTSAGVKL-VEYPLWVTLGRSTPYS
Solyc09g014720.   80 PFGI--GSGCALDPMFEIDCNV-----TTPFIGNIQIYDISDAEMRISNFINTKCYSQTGVLI-QDIPSWITLGTKSPYT
Solyc09g014710.   71 PFGIGLGSGCALDPNFEINCDTDTIDSPTPFIGNIRVYDISDAEMRVSTNINQRCYSSTGRLL-RNDPSWMNLGTSSPYS
consensus         81 ****..*..**.*. ***.*     .  ..*....**..*****.*.*..  ....**...* ...    *.*. .*...**.

Solyc09g014740.  137 FSALNMFTVIGCDETAIMRGDNFRNGCYAFCTNSSGVNVVEGRCMGTGCCQIEIPKGLKYFNTTMDS-VENHTGIWSFNR
Solyc09g014730.  131 FSTLNRFTVVGCDDSAIISSTKFINGCPTTCISSS--HVVDGSCMASGCCQIQIPKGLKNFNTSMQS-SRNHSRIWPFNP
Solyc09g014720.  152 FSTLNRFIVVGCDDGAIVSGNNFANGCPSLCTSTN--DIVKGKCMGFGCCQITIPKGLKFFNTTMVT-TRNHSLIWSFNP
Solyc09g014710.  150 FSTLNRFTVVGCDEVAIIFGGGFANGCPAICINTS--QVTEGSCMGTGCCQITIPKGLRIFNTTMQSSPQNHTGVWSFNP
consensus        161 **.**.*.*.***. **.  .   *.***...*........ .*.**..*****.*****. ***.*..    **...*.**.

Solyc09g014740.  216 CGYAFLGEASRFQFRGEHDLTDNDLRNRILDHVPIVLDWAIGNNLTCGDAQKRDDYACLVNSHCVDSDTGLGGYRCHCDQ
Solyc09g014730.  208 CGYAFLGEASRFQFRGMEDLNDLNFVEKIVNNVPIVLDWAIGN-LTCVEAKKRNDYGCRVNSQCVDSDTGLGGYRCRCNP
Solyc09g014720.  229 CGHSFLGEASRFEFQGIEDLSDVNFANKIRNNVPIVLDWAIGN-LSCVEARKSNDYACLNNSQCVDSDTSLGGYRCSCNS
Solyc09g014710.  228 CGYSFLGEGSRFEFKGLQDLRDLNFKKRILDNVPIVLDWAIGT-LSCVEARKSNDYTCLNNSQCVDSNTGLGGYRCACNP
consensus        241 **..****.***.*.*. ** *.... .*...********** .*.*..*.*..** *..**.**** *.****** *..

Solyc09g014740.  296 GYEGNPYIRPGCSDIDECKNASTCAHYMNCINKPGSYKCYCPKGYTDGGENNGPVCIAT--NSEFPWIKFSVGMGVGFIS
Solyc09g014730.  287 GYEGNPYL--GCRDIDECANPNTNSCEQNCINIPGSYNCSCPQGYTGDGKKNGRGCNAIISNSEFPWIKFSVGMGVGFMS
Solyc09g014720.  308 GYIGNPYIGSGCQDIDECADPNTNSCEKICTNIPGSYNCSCPEGYTGDGRKNGRGCIAPNSNSEFPWIKFSVGMGVGFMS
Solyc09g014710.  307 GYEGNPYIGPGCQDIDECLNPNTNLCEQICINLPGSYNCSCPQGYRGDGRKNGRGCIAPNSNSEFPWIKFSIGMGVGFMS
consensus        321 **.****...**.*****  ...*. ....*.*.****.*.**.** ..*..**..*.*...**********.******.*

Solyc09g014740.  374 LVVGTMLLYFCINKRKLIKNREKFFQQNGGLLLKQQISSKKGGVEATKIFTSDELKKATNNYASDRILGRGGNGIVYKGI
Solyc09g014730.  365 LVLGTTWLYFSFKKRKLMKLREKFFQQNGGFLLKQRISSNEGGVEATKIFTAEELKKATNNYASDRILGRGGNGIVYKGI
Solyc09g014720.  388 LVIGTTWLYFFIKKRKLIKLREKFFQQNGGLLMKQRMSSNEGGVEATKIFTAAELKKATNNYASDRILGRGGNGIVYKGI
Solyc09g014710.  387 LVVGTTWLYFSIKKRKLIKLREKFFQQNGGLLLKQRISSNEGGVGATKVFTAEELKKATNNYASDRILGRGGNGIVYKGI
consensus        401 **.**..***...****.*.**********.*.**..**..*** ***.**..****************************

Solyc09g014740.  454 LPDNRIVAIKKSKIVDENQIEQFINEVLILTQVNHRNVVRLFGCCLEAEVPLLVYEYVSNGTLYEHIHNQNGVPWLSLQN
Solyc09g014730.  445 LPDNRIVAIKKSKFVDENQVEQFINEVLILTQVNHRNVVKLFGCCLEAEVPLLVYEYVSNGTLYEHIHNKNGAPWLSLEN
Solyc09g014720.  468 LSDNRIVAIKKSKFMDEEQVEQFINEVLILTQVNHRNVVRLFGCCLEAEVPLLVYEYISHGTLYEHIHNRNGAPWLSWEN
Solyc09g014710.  467 LPDNRIVAIKKSKFVDEDQIEQFINEVLILTQVNHRNVVRLFGCCLEAEVPLLVYEYVSNGTLYEHIHNQNGAPWLSWKN
consensus        481 *.***********..**.*.*******************.*****************.*.*********.**.****. *

Solyc09g014740.  534 RLRIASETANSLAYLHSSASMPIIHRDVKSSNILLDNGYTAKVADFGASRLVPLDQTRVATLVQGTLGYLDPEYFHTGQL
Solyc09g014730.  525 RLRIASETASSLAYLHSSASMPIIHRDVKSANLLLDDVYTAKVADFGASRLVPLDQTHVATMVQGTLGYLDPEYFHSGQL
Solyc09g014720.  548 RLRVASETASALAYLHSSAQMPIIHRDVKSANLLLDDVYIAKVADFGASRLIPIDQTHLATMVQGTLGYLDPEYFRTSQL
Solyc09g014710.  547 RLRVASETASALAYLHSSAQMPIIHRDVKSANLLLDDVYTAKVADFGASRLIPLDQTHIATLVQGTLGYLDPEYFRLSKL
consensus        561 ***.*****..********.**********.*.***..*.***********.*.***..**.**************. . *

Solyc09g014740.  614 TDKSDVYSFGVVVAELLTGMKPISRDTISDKDKCLVEYFVSSMNKNSLFQILDRRVVREGSLEQLQKIAEIIKRCLHLHG
Solyc09g014730.  605 TEKSDVYSFGVVLAELLTGLKPILKEE-NEKDKCLIDYFILSMNTNNLFQILDRRVVREGSLEQLQKIAELVKGCLSLRG
Solyc09g014720.  628 TEKSDVYSFGVVLAELLTGMKPISKDR-NEEEKNLAEYFVLSMRRNQLFQILDRRVVREGSLEQLQKVAELVKSCLSLHG
Solyc09g014710.  627 TEKSDVYSFGVVLAELLTGIKPISRDK-NNKDKNLAECFILSMRKNQLFQILDRRVVKEGSLEQLQKVAELVKNCLRLHG
consensus        641 *.***********.******.***.....  ..*.*..  *..**..*.*********.*********.**..* ** *.*

Solyc09g014740.  694 EDRPTMKEVAMEIESLRKLTS--LWSNGKEHEDEKEVELTDLYTTPIDSNIGIDNFSGQYPT-SYTNSSIFSGQYNSDSS
Solyc09g014730.  684 EDRPTMKEVAMELESLRKFTKNNPWANGHEDE-ENEDEVKDLYTIDPIDSNADNEFSGQYSSYSPSL-----------I
Solyc09g014720.  707 EDRPTMKEVASELENLRKFTKNNPWANGNGHE-ENEDELSDLYTIPIESNTDIDNFSGQYSSNSYTNSSNFSGQYSSGST
Solyc09g014710.  706 EHRPTMKEVAMKLESLRKFTKNNPWANGHGPE-ENEDESTDLYTIPIDSNTGINKFSGQNSSISNTNTSMFSSLIYTKT-
consensus        721 * ********. .*.***.*....*.**.. *.*.*.* .****..........  ****  .. *  .......    . .

Solyc09g014740.  771 SLLHNTNIPR-------
Solyc09g014730.  751 ---YHTNIPR-------
Solyc09g014720.  786 ---SNTNSPLMKNRRAI
Solyc09g014710.  784 ------NIPR-------
consensus        801 ...    *.*.........
```

Supplementary Figure S1. Multiple sequence alignment of WAK proteins encoded by genes found in the cluster of the genomic region SL2.50ch09:6767500..6819500: Solyc09g014710 (SlWAK2), Solyc09g014720 (SlWAK1), Solyc09g014730 (SlWAK3) and Solyc09g014740 (SlWAK4). Conserved domains identified by making use of different bioinformatics tools (see Material and Methods) are highlighted in colours: In yellow the Ser/Thr kinase catalytic domain (STKc); in red the calcium-binding EGF-like domains (EGF-Ca); in green the cell wall-associated receptor kinase galacturonan-binding Cys-rich domain (GUB-WAKb), and in blue the transmembrane domain.

A

1-MQHYQVALFSFQLPCFMLILTLATAQIIPSNTTSPPTNSTSPPTNATAPAPSPTNTITKAANITKPGCPKQCGNVTVP
79-YPFGIGSGCALDPMFEIDCNVTTPFIGNIQIYDISDAEMRISNFINTKCYSQTGVLIQDIPSWITLGTKSPYTFSTLNR
157-FIVVGCDDGAIVSGNNFANGCPSLCTSTNDIVKGKCMGFGCCQITIPKGLKFFNTTMVTTRNHSLIWSFNPCGHS
232-FLGEASRFEFQGIEDLSDVNFANKIRNNVPIVLDWAIGNLSCVEARKSNDYACLNNSQCVDSDTSLGGYRCSCNS
307-GYIGNPYIGSGCQDIDECADPNTNSCEKICTNIPGSYNCSCPEGYTGDGRKNGRGCIAPNSNSEFPWIKFSVGM
381-GVGFMSLVIGTTWLYFFIKKRKLIKLREKFFQ-413

B

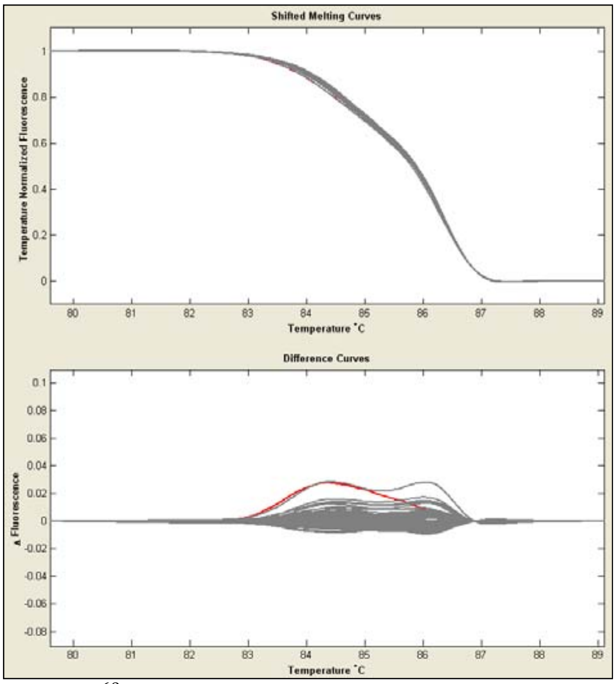

C

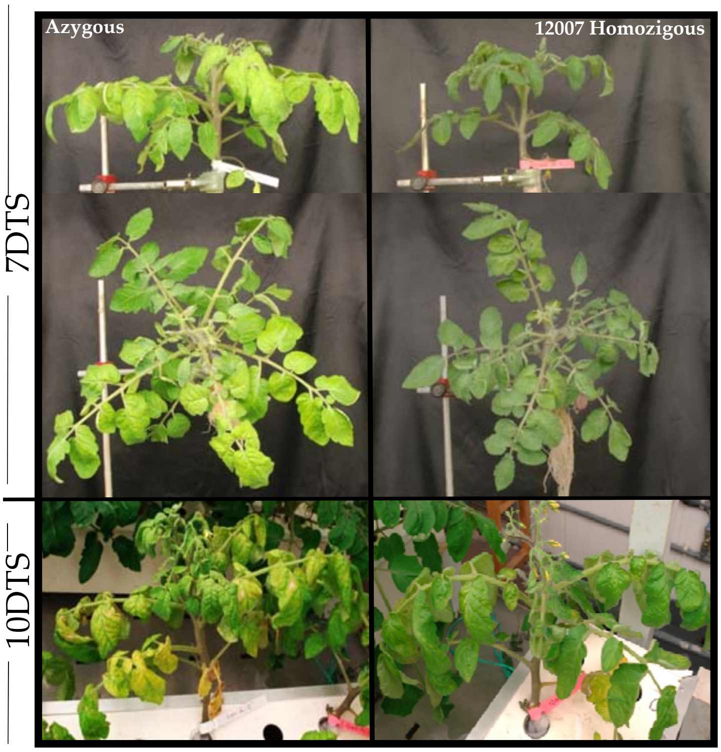

D

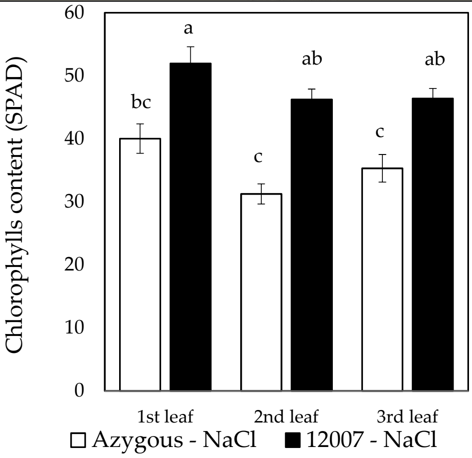

E

	Azygous line	12007 line
Root weight (g)	20.54 ± 1.94	16.13 ± 1.96
Shoot weight (g)	79.60 ± 4.93	**54.11 ± 1.54***
Root water content (g g^{-1}DW)	17.78 ± 1.78	16.50 ± 2.66
Leaf water content (g g^{-1}DW)	14.56 ± 0.90	**11.13 ± 0.33***

Supplementary Figure S2. EMS 12007 line homozygous for the single SNP in *SlWAK1* yielding a truncated protein exhibited lower leaf chlorosis, higher leaf chlorophylls content and lower shoot weight and leaf water content in salt stress condition (200 mM NaCl) compared with 12007 azygous line. Amino acid sequence predicted from the *SlWAK1* gene coding sequence in EMS 12007 line with a premature stop codon in position 414, boxed amino acid in figure 1C (the same color patterns are used for predicted conserved protein domains) (**A**). Output data from HRM analysis (Shifted Melting Curves and Difference Curves) for EMS 12007 line (red color line) (**B**). Phenotypes of azygous 12007 and homozygous 12007 lines after 7 and 10 days of salt treatment (DST) (200 mM NaCl) (**C**). Chlorophylls content in 1st, 2nd and 3rd leaf after 10 DST (**D**). Root and shoot weight, and root and leaf water contents after 10 DST (**E**). Data in **D** and **E** are expressed as mean ± SE (n=5). Asterisks and different letters indicate significant differences according to Student's *t* and Tukey's tests, respectively (p<0.05).

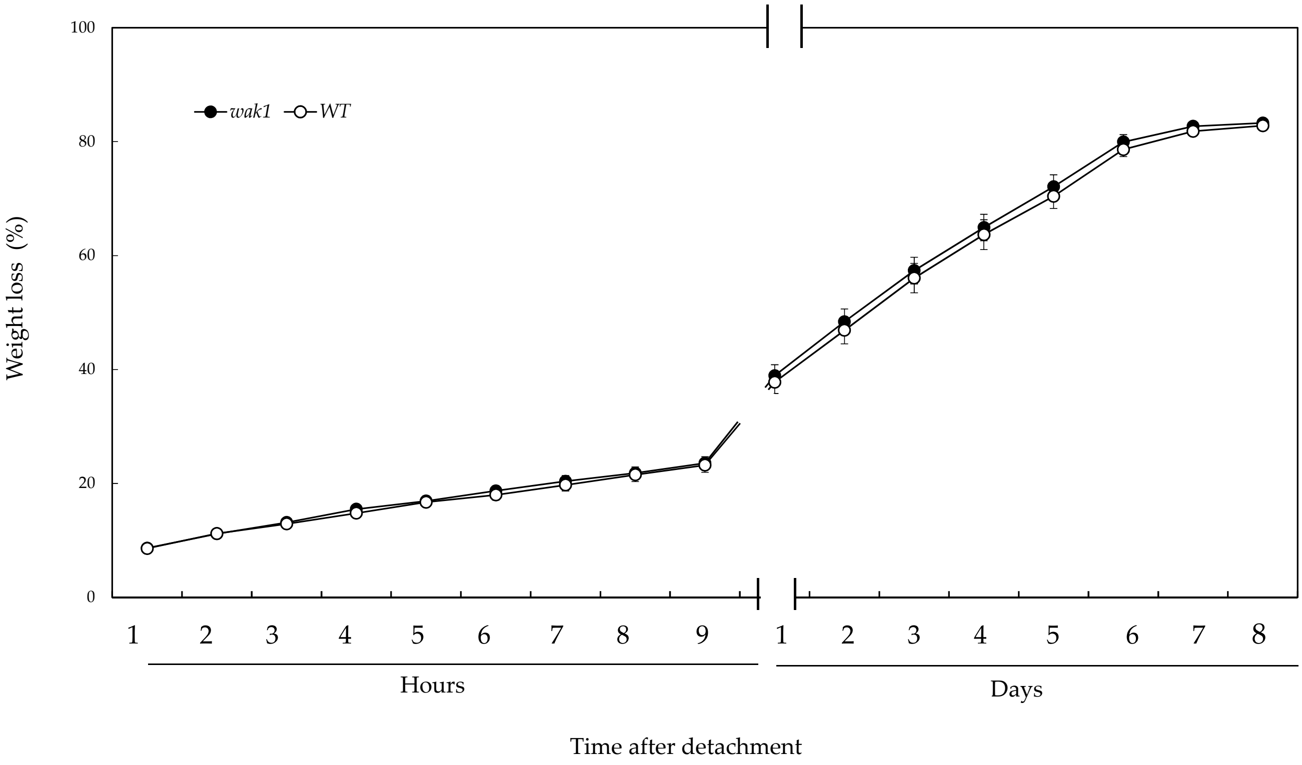

Supplementary Figure S3. Water loss rate measured in detached leaflets coming from the 4th leaf of *Slwak1* and WT plants grown in control. Measures were taken during the first 9 h and afterwards each 24 h (from 1 to 8 days).

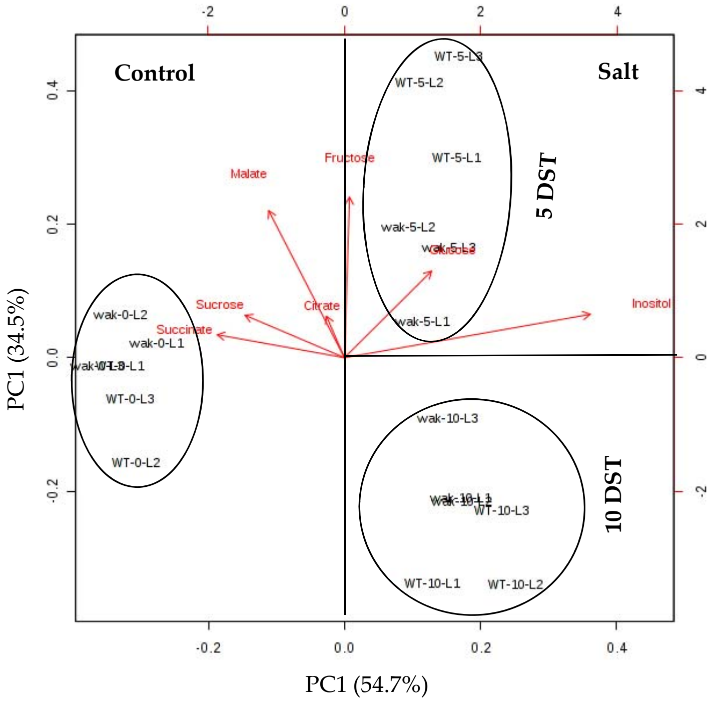

Supplementary Figure S4. PCA-biplot in source leaves of WT and *Slwak1* plants grown at 0, 5 and 10 days of salt treatment (200 mM NaCl).

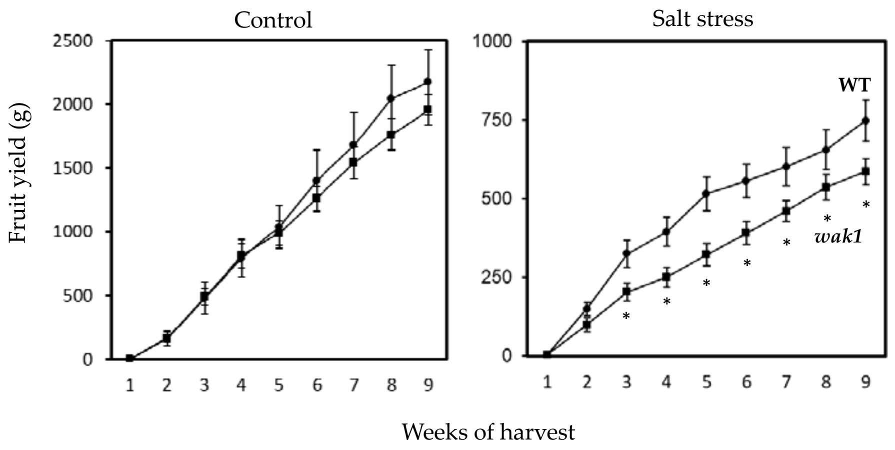

Supplementary Figure S5. *Slwak1* mutant reduces its fruit yield under salt stress. WT and *Slwak1* plants were grown without salt (control) and with salt stress (100 mM NaCl), and red fruits were collected for 9 weeks. Data are expressed as mean ± SE (n=8). Asterisks indicate significant differences between mean values of WT and Sl*wak1* at the same harvest week (Student's *t*-test, p<0.05).

Supplementary Table S1. Expression of *SlSOS1* and *SlHKT1.2* in roots of WT and Sl*wak1* plants grown without salt (control) and with salt (5 days at 200 mM NaCl). Values are the mean ± SE of two independent assays, each with three biological replicates.

	SlSOS1		***HKT1.2***	
	Control	Salt	Control	Salt
WT	1.00	1.40 ± 0.23	1.00	1.64 ± 0.28
wak1	1.18 ± 0.13	1.55 ± 0.13	1.23 ± 0.12	1.56 ± 0.11

Supplementary Table S2: Primers used (* in this study)

List of primers used in qPCR:

Gene	*Locus*	*Sense (5′-3′)*	*Antisense(5′-3′)*	*Ref*
Wall Associated Kinase genes				
SlWAK1	Solyc09g014720	GGTTGTGATGACGGTGCTAT	CAGCAACCAAAACCCATACA	*
SlWAK2	Solyc09g014710	TTTTCAACACAACGATGCAAA	TCAAATCGACTACCTTCACCA	*
SlWAK3	Solyc09g014730	TGTGGGAACATCACAATTCC	TGTTGGAGTCCCTGTCGATT	*
Sodium transporter genes				
SlSOS1	Solyc01g005020	TCGAGTGATGATTCTGGTGG	GACGCCTTTCCACACTCTGAT	[13]
SlHKT1.2	Solyc07g014680	TGAGCTAGGGAATGTAATAAACG	AGAGAGAAACTAACGATGAACC	[13]
Sucrose metabolism genes				
SlTIV1	Solyc03g083910	AAACTCCGCCTCTCGTTACA	CAAAAGGAAAACGGAGAGGA	*
SlLIN6	Solyc10g083290	TTCTTCTCTTTGGGCTTTGC	TGGACCTTGCTAGCATCAAC	*
SlINH	Solyc12g099200	GTATGCCAGAAGCATTAGAAGCA	GCATCACCAGAAGAACCAACC	[11]
Reference (house-keeping) gene				
LeEF1α	Solyc06g005060	GACAGGCGTTCAGGTAAGGA	GGGTATTCAGCAAAGGTCTC	[13]

List of primers used in PCR amplification, genotyping and Anchor-PCR:

Gene	*Sense (5′-3′)*	*Antisense (5′-3′)*		*Ref*
Presence of T-DNA				
NptII	ATCCATCATGGCTGATGCAATGCG	CCATGATATTCGGCAAGCAGGCAT		[26]
Genotyping analysis (primer sequence 5′-3′)				
WAK1-F	*WAK1-R*	*ARB-2*		*
TCCCACCACTCCTATCTTCG	GTAGGCATGGCCAACCAATA	GGTCTTGCGAAGGATAGTGG		
Anchor-PCR analysis (primer sequence 5′-3′)				
Ad1	*Ad2*	*Ad3*		*
CTAATACGACTCACTATAGGC	CTATAGGGCTCGAGCGGC	AGCGGCGGGGAGGT		
ARB-1	*ARB-2*	*ARB-3*		*
ACAGTTTTCGCGATCCAGAC	GGTCTTGCGAAGGATAGTGG	CTGGCGTAATAGCGAAGAGG		
ALB-1	*ALB-2*	*ALB-3*		*
TTGGCGTGTCAGCGTATCTA	ATCGGTCTCAATGCAAAAGG	ATAATAACGCTGCGGACATCTAC		

List of primers used in PCR (TILLING Strategy):

Targeting Induced Local Lesions IN Genomes analysis (primer sequence 5′-3′)			
Amplicon 1	*Amplicon 2*	*Amplicon 3*	*Ref*
Solyc09g014720_F1	Solyc09g014720_F2	Solyc09g014720_F3	*
AAACACAGGTATGGGAGTTGG	ATGGACGAGGAACAGGTTGA	GCTACAATGGTTCAAGGGACA	
Solyc09g014720_R1	Solyc09g014720_R2	Solyc09g014720_R3	
TCAACCTGTTCCTCGTCCAT	TGACTTGTCCGAAAGTATTCAG	TGATAATTCATCTTCGTTCTCTTCA	
